# A Review and Evaluation of the Data Supporting Internal Use of *Helichrysum italicum*

**DOI:** 10.3390/plants10081738

**Published:** 2021-08-23

**Authors:** Katja Kramberger, Saša Kenig, Zala Jenko Pražnikar, Nina Kočevar Glavač, Darja Barlič-Maganja

**Affiliations:** 1 Faculty of Health Sciences, University of Primorska, 6310 Izola, Slovenia; katja.kramberger@fvz.upr.si (K.K.); sasa.kenig@fvz.upr.si (S.K.); zala.praznikar@fvz.upr.si (Z.J.P.); 2 Faculty of Medicine, University of Ljubljana, 1000 Ljubljana, Slovenia; 3 Faculty of Pharmacy, University of Ljubljana, 1000 Ljubljana, Slovenia; nina.kocevar.glavac@ffa.uni-lj.si

**Keywords:** *Helichrysum italicum*, biological activity, internal use, clinical studies

## Abstract

*Helichrysum italicum* is a Mediterranean plant with various pharmacological activities. Despite extensive reports on the bioactivity of the plant, its clinically studied applications have not yet been reviewed. The aim of our study was to gather information on the internal use of *H. italicum* and its bioactive constituents to determine its efficacy and safety for human use. We reviewed research articles that have not been previously presented in this context and analyzed relevant clinical studies with *H. italicum*. Cochranelibrary.com revealed six eligible clinical trials with *H. italicum* that examined indications for pain management, cough, and mental exhaustion. Although the efficacy of *H. italicum* has been demonstrated both in in vitro tests and in humans, it is difficult to attribute results from clinical trials to *H. italicum* alone, as it has usually not been tested as the sole component. On the other hand, clinical trials provide positive information on the safety profile since no adverse effects have been reported. We conclude that *H. italicum* is safe to use internally, while new clinical studies with *H. italicum* as a single component are needed to prove its efficacy. Based on the recent trend in *H. italicum* research, further studies are to be expected.

## 1. Introduction

Plants have a long history of use in medicine and have been used by all cultures or ethnic groups throughout history to improve human health [1]. They are considered to be the oldest form of medicine known to humankind but are, on the other hand, also an important source of modern medicines and govern synthetic drug development [1,2]. According to the World Health Organization (WHO), 70–95% of the world’s population rely on traditional medicine for their primary health care [2]. This is especially true for the Mediterranean countries, where plants play a vital role in the diet habits, and sometimes there is no clear dividing line between food and medicinal plants, particularly in indigenous and local traditions [3]. 

**Figure 1:**
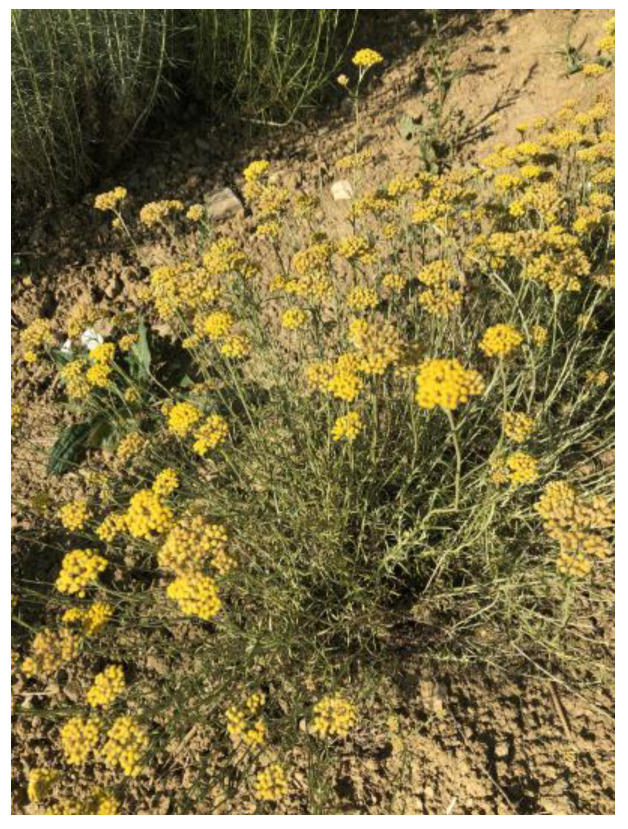
Cultivated *Helichrysum italicum* plant in flowering stage (Photo: Katja Kramberger).

The plants belonging to the genus *Helichrysum* (family Asteraceae) are known as everlasting flowers and are widely used in traditional medicine worldwide [4]. The plant species of this genus typically have inflorescences of a bright yellow color [5], which retain their form and color when dried, hence the name “everlasting” or “immortal” [4]. The stems are woody at the base and can reach 30–70 cm in height. The plant is well adapted to environments that lack water as it naturally grows on alkaline, dry, sandy and poor soil at the altitude from the sea level up to 2200 m [6]. The *Helichrysum* Miller genus includes more than a thousand of taxa, among them the most well-known and studied species are *Helichrysum italicum* (Roth) G. Don [7] (Figure 1), *Helichrysum stoechas* (L.) Moench [8], and *Helichrysum arenarium* (L.) Moench [9]. *H. italicum* and *H. stoechas* are distributed throughout the Mediterranean [10] but are especially characteristic to Adriatic region [11] and Iberian Peninsula [12], respectively, whereas *H. arenarium* is mostly found in the Central Europe [9,10]. Despite the long tradition in treatment of various disorders for all three before-mentioned species, their traditional use for treating digestive problems (e.g., fullness and bloating) has been approved by the WHO and the European Medicines Agency (EMA) for the species *H. arenarium* alone [8]. Nevertheless, *H. italicum* has been recently investigated quite extensively, especially in the Balkan countries. The focus of this review article will be from herein after on *H. italicum* only. Among the *H. italicum* species, there are also several subspecies (abbreviated ssp.), which are difficult to distinguish due to a strong polymorphism in morphology [10]. The explanation of *H. italicum* classification is beyond the scope of this review article, but the name of subspecies is included, if available in the referenced article.

Characteristic yellow fade-resistant inflorescences (seen in Figure 1) as well as vegetative aerial organs of *H. italicum* are a treasury of bioactive secondary metabolites that result from the plants’ adaptation to this challenging environment. Apart from the volatile terpenes present in essential oils, absolutes and supercritical CO_2_ extracts, *H. italicum* is also very rich in phenolic compounds, which are recognized as health promoting agents due to antioxidant properties they exert and their probable role in the prevention of various diseases associated with oxidative stress, such as cancer, cardiovascular and neurodegenerative diseases [13]. The health-beneficial potential of *H. italicum* has been reported in ethnopharmacological surveys and supported by numerous in vitro and in vivo experiments [7]. In the Greek-Roman system of medicine, *H. italicum* was used as an anti-inflammatory and anti-infective plant, and both uses are still well rooted in traditional medicine today [14]. Antunes Viegas et al. [7] emphasized that, in contrast to animal studies, there is a severe lack of clinical studies investigating the effects of the *H. italicum* extracts, which undermines the possibility of validating the traditional uses of this plant. One of the reasons why clinical and other comprehensive studies with herbal products are scarce probably lies in the difficulty of interpreting the results of such studies [2].

The aim of our study was to gather all information reported in the literature on internal applications of *H. italicum* or its bioactive constituents in humans to support the efficacy and safety of *H. italicum* preparations for human use. We reviewed studies that have not been outlined in this context before and analyzed relevant clinical trials with *H. italicum* as the main or one of the studied components.

## 2. Methodology

Research and review articles were searched via online databases including PubMed and Scopus until February 2021. Clinical trials on *H. italicum* and its constituents were searched through Cochrane Library (https://www.cochranelibrary.com/) and ClinicalTrials (https://clinicaltrials.gov/) (last accessed on 2 May 2021).

The reviewed literature on *H. italicum* use is presented in thematic sections; ethnopharmacological surveys and non-clinical research articles (Section 3), human clinical observations (Section 4), clinical trials (Section 5) and recently published articles (Section 6). The first section briefly reviews the use of *H. italicum* in traditional medicine, as well as the most prominent in vitro and in vivo scientific experiments. Section on human clinical observation summarizes the experiments in humans, which were not registered as clinical trials. Clinical trials section is divided in clinical trials with *H. italicum* (Section 5.1) and trials with isolated compounds commonly found in *H. italicum* (Section 5.2). Lastly, recently published articles on *H. italicum* are presented to provide insight into the current trend of *H. italicum* research.

## 3. Traditional Uses and Scientific Data

Ethnopharmacological surveys on *H. italicum,* summarized in the study by Antunes Viegas et al. [7], show that the most frequently reported traditional uses are related to respiratory, digestive and skin inflammatory conditions. Depending on the application, *H. italicum* preparations are administered via inhalation, ingestion or topically. Other therapeutic applications include wound healing and antimicrobial uses, as well as gall and bladder disorders and analgesic use. Common types of preparations are mostly infusions and decoctions, for both oral and external use, followed by vapors, juices and powders [7]. Several in vitro as well as in vivo studies have confirmed biological activity of compounds isolated from *H. italicum* or its fractionated extracts, whereas for some indications such as digestive non-inflammatory disorders, pain in the gastrointestinal tract, alopecia, helminthic infections, and sleeplessness, scientific validation is still missing [6]. Moreover, studies investigating crude extracts, especially aqueous extracts, which are the most commonly used in traditional medicine, are rather scarce. 

A large variety of *H. italicum* extracts can be prepared, and the resulting products differ in their chemical composition, yet the main compound classes are terpenes and phenolics. A systematic review of *H. italicum* bioactive compounds with regard to extraction procedure used for their isolation was conducted by Maksimovic et al. [15] and for the most characteristic compounds this information is summarized in Table 1. Research on *H. italicum* regained interest in the 1990s with the studies by Facino et al. [16,17], Pietta et al. [18,19] and by Zapesochnaya et al. [20,21] on isolation and identification of bioactive substances of Italian *H. italicum*. Numerous studies were performed in the following decades, in which additional bioactive constituents were isolated and in vitro and in vivo tests performed to support their bioactivity [14,22,23,24,25,26,27,28,29,30,31,32,33,34,35,36,37,38,39,40,41,42]. Nostro et al. [22,23,24,25] investigated anti-cariogenic potential of *H. italicum* diethyl ether and ethanolic extracts, which is probably attributed to flavonoids. Studies by Sala et al. [26,27,28,29] dealt with a class of acetophenone compounds and flavonoids pinocembrin, gnaphallin and tiliroside, isolated from *H. italicum*, and tested their anti-inflammatory action in mice. Noteworthy are also the studies by Appendino et al. [14], Rosa et al. [33,34] and Bauer et al. [35], who discovered and investigated a main anti-inflammatory compound present in *H. italicum*, arzanol. Its anti-inflammatory effects have also been proved in vivo [35]. Arzanol’s activity and its mechanism of action are summarized in review by Kothavade et al. [43]. The following studies are worth mentioning due to validating traditional uses at in vivo level. Rigano et al. [39] proved that ethanol extract elicited antispasmodic action in the isolated mouse ileum and inhibited transit preferentially in the inflamed gut. The suitability of the traditional use of *H. italicum* ssp. *italicum* flowers for intestinal diseases was thereby confirmed. De la Garza et al. [38] showed that methanol-water extract decreased blood glucose levels and reduced postprandial glucose levels, as well as improved hyperinsulinemia in a dietary model of insulin resistance in rats. Furthermore, Pereira et al. [42] investigated anti-diabetic activity of water-based preparations (infusions and decoctions) of *H. italicum* ssp. *picardii* via inhibitory activity towards α-glucosidase and found moderate effects. Although diabetes is not one of the conditions mentioned in traditional medicine of *H. italicum*, this study opened another possible application—treatment of metabolic syndrome. 

In recent years, increased interest for *H. italicum* was also observed in many Southern European countries, predominantly due to *H. italicum* essential oil and its use in the perfume and cosmetic industry. This topic is reviewed by Ninčević et al. [10], who also focused on taxonomic classification and morphological characteristics arising from genetic diversity, in addition to bioactive compounds of *H. italicum* and their biological activity.

## 4. Human Clinical Observations

Despite several review articles on *H. italicum*, the following clinical observations are rarely mentioned. Systematic clinical studies on the anti-inflammatory properties of *H. italicum* were already carried out by Leonardo Santini, an Italian physician, in the 1940s. Despite the promising results, these investigations were largely overlooked at that time, but were later recognized as relevant for studies on anti-inflammatory activity of *H. italicum* [14]. The clinical experiments performed by Santini [49], Benigni [50], Vannini [51] and Campanini [52] are described in Italian literature and summarized in the article by Appendino et al. [53]. These observations are presented in Table 2 along with additional studies by Facino et al. [16], Voinchet and Giraud-Robert [54], and a very recent one by Granger et al. [55]. These studies mainly cover treatments of respiratory and dermal conditions.

## 5. Registered Clinical Trials

### 5.1. H. italicum Herb

**Figure 2:**
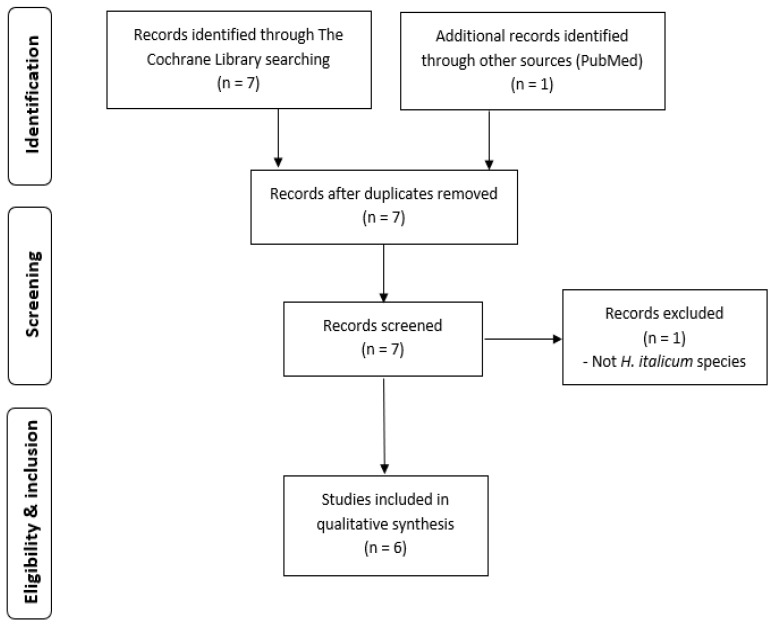
Flowchart of the trials’ selection process. n—number of records/studies.

To date, no review of clinical trials including *H. italicum* has been conducted. Searching the Cochrane Library for the term “Helichrysum” in Title Abstract Keyword (Word variations have been searched) resulted in seven hits (https://www.cochranelibrary.com/, accessed on 4 January 2021). An additional record was identified through PubMed database. After inspection, one duplicate record was identified, and another one was excluded based on an inappropriate *Helichrysum* species investigated. Other six records addressed *H. italicum* alone or in combination with other herbs. The indications studied were pain (chronic prostatitis, post-surgical pain), cough and mental exhaustion. Consequently, diverse dosage forms were used: granules, syrup, inhalation preparation and suppositories. All relevant records were included in the qualitative synthesis, although few were missing full-text articles. The above-described process of literature search and article selection is shown in Figure 2. No meta-analysis could be performed, as there were too few trials published, and the presented trials possessed too much heterogeneity both in studied conditions and in the formulations tested.

The reviewed clinical trials on *H. italicum* are summarized in Table A1 and described in more detail in the following paragraphs. The first trial, chronologically, was performed by Aboca S.p.A., an Italian company focused on innovative products based on natural substances. According to the EU clinical trials register record [56], two commercial products in the form of granules for oral suspension were administered as a pain treatment to adult patients with post-surgery pain. Freeze-dried extracts of *H. italicum* flowering tops and *Salvia officinalis* (sage) leaves were parallelly compared against placebo. Although trial status is “completed”, the results are not available in the databases, and no articles have been published. We also tried contacting the company directly but were unsuccessful. 

In the trial by Galeone et al. [57], *H. italicum* was incorporated in the medical device Proxelan^®^ (Sala Bolognese, Italy) suppositories along with other plants: *Boswellia serrata*, *Centella asiatica* (Asiatic pennywort) and *Cucurbita pepo* (pumpkin) seeds. Altogether sixty subjects with bacterial and non-bacterial prostatitis were divided in two groups, one receiving antibiotic treatment and the other receiving antibiotics together with Proxelan^®^ suppositories. Minor side effects were observed, but they did not cause trial interruption in any case. From a microbiological point of view, Proxelan^®^ treatment was not better than antibiotics alone (*p* = 0.46). However, the combination of antibiotics and Proxelan^®^ improved both symptoms associated to chronic prostatitis and urinary symptoms, which were two-fold decreased compared to control group after two months following the intervention (*p* = 0.028). The trial provides some relevant information on the safety since the rectal application can also have systemic effects. Conclusions regarding efficacy are not as straightforward, as *H. italicum* was not a single plant in that formulation. 

Additional trial with the same product has been published more recently [58]. This time, authors aimed to investigate the effects of Proxelan^®^ monotherapy on the pain symptoms of patients with a clinical diagnosis of chronic abacterial prostatitis or chronic pelvic pain syndrome. Proxelan^®^ suppositories were prescribed to thirty male patients for a month with a daily dosage of one suppository at bedtime. Subjective pain relief was obtained in all the patients (*p* = 0.04). Urinary symptoms, investigated by questionnaire, decreased significantly (*p* = 0.04), and the quality of life improved (*p* = 0.04). Further seminal investigations were performed on a subset of patients. In a one-month follow-up, leukocytospermia decreased substantially or disappeared, IL-6 decreased by 11.55%, while IL-8 values did not show significant variation. The sperm motility increased by 17.3% and spermatozoa concentration remained unchanged. The medical device showed efficiency in pain reduction, as well as in improvement of semen quality by addressing the inflammatory component of this condition. This trial thus confirmed that Proxelan^®^ monotherapy can be successfully used without antibiotics combination treatment to obtain comparable clinical outcomes in patients with chronic prostatitis or chronic pelvic pain syndrome symptoms. However, the results obtained should be investigated on a larger cohort of patients in randomized controlled trials.

Another trial was published by Varney and Buckle [59], who investigated the effect of *H. italicum* essential oil on mental exhaustion and moderate burnout. Patients were given a personal inhaler with mixture of essential oils (peppermint, basil, and everlasting) or placebo (rose water), which they administered themselves three times in each nostril every hour of the working day (approx. seven times per day) for the duration of five days. According to the authors, this mixture contained two stimulant essential oils to address the fatigue, and one balancing essential oil to address the anxiety. In aromatherapy, *Mentha* x *piperita* (peppermint) essential oil is used to increase alertness and mental clarity, and *Ocimum basilicum* ct *linalol* (basil) essential oil to reduce mental fatigue and achieve antidepressant properties. *H. italicum* essential oil is known for its calming and soothing properties. The participants self-assessed their feelings via questionnaire three times per day in the intervention week, as well as one week before and after. Both groups reported reduction in perception of mental exhaustion or moderate burnout, whereas for the aromatherapy group, reduction was two times greater. Although the results were encouraging, they may not be generalizable due to the small population tested and due to some reported inconsistency in the administration. 

The trial led by Cohen et al. [60] was by far the most extensive and multicentered. It included 150 children over four pediatric clinics in Israel. The purpose was to determine if there is comparable efficacy between mucolytic substance carbocysteine and a protective cough syrup (Grintuss^®^, Sansepolcro, Italy) based on natural ingredients on children’s cough due to upper respiratory tract infections, such as the common cold. Mucolytic agents have been shown to be helpful, but serious side effects have been reported, and the use has been prohibited for children under two years of age. Therefore, safer alternatives for cough management, which function via other mechanisms such as irritated pharynx mucosa protection, were explored. Grintuss^®^ syrup contained a combination of specific substances such as resins, polysaccharides, saponins, flavonoids and sugars derived from *Grindelia robusta* (gumweed), *Plantago lanceolata* (ribwort plantain), *H. italicum*, and honey. The protective effect of the syrup on the mucosa of the upper respiratory tract was exerted by a local mechanical barrier (limiting cough stimuli with a non-pharmacological approach, but with an indirect anti-inflammatory action), as well as by radical scavenging action. A survey was conducted among parents on four consecutive days, where treatment was Grintuss^®^ or Mucolit^®^ (Kiryat Malachi, Israel), with single-blinded randomization, 3 times per day for 3 days. Both syrups were well tolerated, and the cough was alleviated. There was a significantly better result throughout for Grintuss^®^ (*p* < 0.05) after one day for all the main outcome measures (cough frequency, cough severity, bothersome nature of cough, and sleep quality for both a child and a parent). The trend for improvement over the four days was steeper for Grintuss^®^ (*p* < 0.05) for all cough parameters. Although both syrups were effective and safe treatments for children over two years of age, Grintuss^®^ appeared to produce faster (first night) and more effective response (over four days of treatment) as to clinical cough symptoms. This trial reveals important information on the safety of *H. italicum* even for young children. 

In the trial by Canciani et al. [61], Grintuss^®^ syrup was compared to a placebo syrup in young children suffering from persisting cough. Both syrups were taken in four doses per day for eight days. None of the patients discontinued the trial for adverse events, or other safety reasons. The authors, however, state that Grintuss^®^ should not be used in case of known hypersensitivity to the components of the medical device, but no other contraindications have been registered. It is worth mentioning that at the time of the research, Grintuss^®^ syrup has been on the market for more than ten years, registered as a medical device (class IIa), during which the post-marketing surveillance system, in compliance with Directive 93/42/EC, has not registered incident or side effects related to the medical device, which further supports the safety of this device.

A similar trial by Calapai et al. [62] investigated the effect of KalobaTuss^®^ (Egna–Neumarkt, Italy) syrup in children with persisting cough. This product contained *H. stoechas* as a component, therefore the record was not evaluated further. However, as the *H. stoechas* is closely related to *H. italicum* [7], these findings might also be relevant.

### 5.2. Individual Bioactive Substances

Several factors, such as the growing conditions, drying, storage and extraction procedure, can greatly affect quality and the composition of an herbal preparation and hence its therapeutic outcome [63]. In modern phytotherapy and traditional medicine, mostly extracts with complex chemical composition are used, rather than isolated substances, favoring occurrence of synergistic effects and polyvalent activity. It needs to be stressed out, that also less abundant compounds can be potent. Consequently, identifying the active constituent in many herbal extracts has often proved to be difficult [64]. Several bioactive compounds were previously isolated from *H. italicum* and their activity investigated (already discussed in Section 3). Pereira et al. [42] investigated the composition of infusions and decoctions of *H. italicum* ssp. *picardii* and established that the main compounds were chlorogenic and quinic acids, dicaffeoylquinic acid isomers and flavonoid gnaphaliin A. According to Karača et al. [65], the most abundant phenolic compounds present in the water extract prepared from commercially available *H. italicum* flowers, were caffeic acid, chlorogenic acid, and its derivatives. Kramberger et al. [66] found that caffeoylquinic acids and pyrones were the most prevalent compounds in *H. italicum* ssp. *italicum* water extracts. Composition of the essential oils, on the other hand, is completely different. In essential oil volatile terpenes predominate to the contrary of polar and semi-polar phenols, which are common in water-based preparations and organic solvent extracts [15]. Some terpenes can also be extracted with non-polar organic solvents or supercritical fluids, but this extraction procedure deviates from the traditional methods of preparations. In the following sub-section, clinical trials on the most relevant individual substances that are confirmed to be present in *H. italicum* water and hydroalcoholic extracts are briefly described.

#### 5.2.1. Phenolic Acids

In contrast to clinical trials evaluating *H. italicum* extracts of a whole plant, trials on isolated substances are more numerous. One of the most studied isolated substances found in *H. italicum* is chlorogenic acid, an ester of caffeic and quinic acid. It is a widely distributed natural compound with many important activities. *In vitro* and in vivo studies have found that the main pharmacological effects of chlorogenic acid are antioxidant, anti-inflammatory, antibacterial, antiviral, hypoglycemic, lipid lowering, anti-cardiovascular, antimutagenic, anticancer and immunomodulatory [45]. 

In the Cochrane Library, there are 160 Trials matching “chlorogenic acid” in Title Abstract Keyword (Word variations have been searched) (https://www.cochranelibrary.com/, accessed on 4 January 2021), and among them, 71 have been published in the recent four years [67]. The trials mostly investigated effects on cardiovascular system, weight loss, chronic inflammatory diseases, cognition, and lung cancer as well as bioavailability. Chlorogenic acid has been investigated alone or as the main component of some dietary supplements (i.e., green coffee extract). As this ubiquitous phenolic acid is present in largest quantities in coffee [68], which is a widely consumed beverage, action in the *H. italicum* should not pose health concerns. 

Similarly, caffeic acid—a very common phenolic acid with antioxidant, anti-inflammatory and anticarcinogenic activity [44], is also a well investigated compound (68 registered trials, accessed on 17.3.2021) [69]. Trials include effects on esophageal cancer, non-alcoholic fatty liver disease, photoprotection, immune thrombocytopenia, and bioavailability studies.

5.2.2. Flavonoids

Pinocembrin is a well investigated flavonoid of *H. italicum* with demonstrated anti-inflammatory action in vivo [29]. To date, there are only two registered clinical trials on pinocembrin, and both have investigated its neuroprotective effect. Pinocembrin was injected into patients with ischemic stroke [70], and in another trial by Cao et al. [71], pharmacokinetics and safety of pinocembrin injection was investigated. When administered intravenously to healthy adults, pinocembrin was well tolerated up to 120 mg/d. Furthermore, no major safety concerns were identified that would preclude further clinical development of pinocembrin injection.

Quercetin is a versatile antioxidant known to possess protective abilities against tissue injury induced by various drug toxicities [46]. It is present in over twenty plants, in *H. italicum* mostly in the form of various glycosides. There are over 497 registered trials in Cochrane Library (https://www.cochranelibrary.com/, accessed on 17 March 2021) investigating versatile interventions [72]. These include effects on the vascular system (cerebral blood flow, vascular function, blood pressure, thalassemia), inflammation (sarcoidosis, asthma, chronic obstructive pulmonary disease), sex hormone disorders (prostate cancer, prostatitis, polycystic ovary syndrome and estrogen deficiency), metabolic disorders (dyslipidemia, glucose absorption, non-alcoholic fatty liver disease), performance (neuromuscular function, endurance, recovery) and other (immune response, stroke, myocardial infarction, hyperuricemia and oral mucositis).

Naringenin is a commonly found flavonoid in citrus fruits but is also found in its glycoside forms in *H. italicum*. Several biological activities have been ascribed to this phytochemical, above all cardioprotective action is the best investigated clinically [47]. Its effects on liver markers and blood pressure or on metabolic rate, insulin sensitivity and blood glucose have been studied in patients with non-alcoholic fatty liver disease or diabetes, respectively [73]. In the trial by Rebello et al. [74] on safety and pharmacokinetics of naringenin consumption, naringenin proved to be safe in healthy adults (up to 900 mg), and serum concentrations were proportional to the dose administered.

Clinical trials have been performed with luteolin as well. Effects on obesity and cardio-metabolic risk factors in metabolic syndrome and on memory and behavior in children with autism were investigated [75]. In addition, its effect on exercise performance was also investigated.

#### 5.2.3. Other Compounds

A triterpene ursolic acid, is one of the non-polar compounds of *H. italicum*, that has been isolated from acetone [37] and methanol extracts [26]. Although it possesses anti-inflammatory, anticancer, antidiabetic, antioxidant and antibacterial effects, its bioavailability and solubility limit its clinical application [48]. Its activity has been investigated in patients with metabolic syndrome and on muscle function. Furthermore, ursolic acid was also injected to patients with solid tumors, where it was shown that ursolic acid liposome does not accumulate in the body. The administration was tolerable, had manageable toxicity, and could potentially improve patient remission rates [76]. 

Lastly, we want to mention also arzanol, which is probably the most characteristic compound for *H. italicum*. Although extensively investigated in vitro and in vivo, to date, no clinical trial has been registered.

## 6. Recent Advances in *H. italicum* Studies

Concurrently with the clinical studies, other publications from the past three years were also evaluated. While the majority of the published articles is still focused on *H. italicum* essential oil, quite some of the recent research has been devoted to minimizing waste from the production process and herewith to follow emerging sustainability and upcycling approaches. Essential oil is produced preferentially from the flowerheads, while the rest of the plant, which contains significant amounts of secondary metabolites and could be used for extract production, is left in situ [53]. Dzamic et al. [77] investigated wastewater extracts of *H. italicum* produced after distillation. The highest phenolic concentration was measured in deodorized aqueous extract. The deodorized aqueous extract also possessed the highest antioxidant activity, followed by deodorized methanol extract, while essential oil had the lowest radical scavenging activity. Addis et al. [78] investigated wastewater extracts of aromatic plants, among them also *H. italicum*. They determined that water decoction not only retains antioxidant activity, but is also effective in wound healing, as it promotes tissue re-establishment after environmental stress exposure. Environmental topics continue to arise as Pilić and Martinović [79] investigated effect of *H. italicum* macerate on the corrosion of copper in simulated acid rain solution and Eksi et al. [80] assessed *H. italicum* as a green roof substrate.

Recently, detailed chemical composition and antioxidant activity of hydroalcoholic and water extracts has been evaluated and compared by Kramberger et al. [66]. In addition, further functional studies with water extracts (infusions) have been performed on cell models and gene expression of oxidative-stress related genes has been carried out [81]. In this comparative study, two morphologically distinct *H. italicum* subspecies were compared with a recognized medicinal plant of *H. arenarium*, on a genetic, chemical, and functional level. Both *H. italicum* subspecies exhibited superior antioxidant activity in vitro as well as cytoprotective activity. As it has been emphasized before [7], genetic or morphological description of the plant material used is often lacking in studies, probably due to the difficult characterization of *H. italicum*, arising from great diversity of the species and disunited classification keys available. In the recently published study by Baruca Arbeiter et al. [82], a set of new microsatellites as DNA markers was developed, which will serve for selection of most promising genotypes for propagation and their implementation in agricultural production. 

## 7. Critical Perspective on Safety and Efficacy

*H. italicum* toxicity has been investigated in some in vitro studies, but in general, this information is scarce. Pereira et al. [42] investigated cytotoxicity of *H. italicum* ssp. *picardii* tisanes towards different mammalian cell lines: hepatocarcinoma (HepG2), microglia (N9) and bone marrow stromal (S17) cell line. The extracts in the tested concentration of 100 µg/mL and after 72 h of exposure had low toxicity, with cell viability values similar or higher than those obtained for green (*Camellia sinensis*) and red bush (*Aspalathus linearis*) teas, which suggest that these aqueous extracts can be regarded as non-toxic beverages. Kramberger et al. [81] evaluated cell viability on lymphoma cell line (U937), adenocarcinoma cell line (Caco-2) and primary colon fibroblasts (CCD112CoN) after exposure to *H. italicum* infusions. Concerning U937 cells infusion was not toxic up to 5% *v/v* concentration, whereas for Caco-2 it was toxic at 1% *v/v*. Interestingly, higher concentration (2% *v/v*) was toxic for CCD112CoN cells, than for cancerous cell line Caco-2. Genotoxic activity of *H. italicum* has been evaluated by Nostro et al. [24], where diethyl ether extract showed no DNA-damaging activity at concentrations up to 2000 µg/disc. Some potential cytochrome P450 enzyme interactions have been discussed by Antunes Viegas et al. [7], more specifically for flavonoid tiliroside. It should be emphasized that the concentrations that are achievable via oral route in vivo may not be sufficient to cause medical important interactions due to low bioavailability. Such interactions are even less likely to occur when administering traditional preparations, where effect of one minor compound usually does not prevail. From the safety perspective, essential oils are potentially more problematic, as they are more concentrated mixtures. In this case, allergic reactions in hypersensitive individuals can occur [83]. 

From the current review of the clinical trials, no adverse events that could be attributed to the tested herbal products, were reported. Very importantly, the review also included trials on a large number of young children. Furthermore, *H. italicum* has a long traditional use and several products containing *H. italicum* are already on the market. However, the products are mostly not registered as therapeutics, but rather as dietary supplements or cosmetics. These products include oral supplements developed to favor venous circulation or cough treatment, while cosmetic products, claim the calming and antimicrobial properties of *H. italicum* essential oil incorporated in their formulas [7]. Appendino [84] strongly believes that the former focus on *H. italicum* essential oil and its application in cosmetics, should be turned to development of novel ingredients for medicinal and health-food products. The use of herbal products in general is very complex; apart from medicines, food supplements and cosmetics, botanicals could be marketed also as food (for example spices or herbal teas) or as medical devices, when the plant product can demonstrate a sole mechanical and non-pharmacological action, such as protection of mucous membranes or skin cooling/warming effect [85]. As these products are not proposed as treatments of diseases, demonstration of their clinical profile is not legally required [86]. Nevertheless, consumers usually choose the ones that better respond to their health needs, often ignoring the fact that diverse marketing categories imply profound differences in terms of manufacturing processes, chemical composition, quality controls, and studies of efficacy [85]. Based on these data all together, it can be concluded that *H. italicum* in the orally acceptable formulations is safe to use but would need further evaluation in the case of consideration as an herbal medicine with well-established use.

Efficacy of *H. italicum* has been established several times both in in vitro tests and in humans; first in human clinical observations and more recently also in clinical trials. Although, there are proper clinical trials that demonstrated its tested efficacy, findings are difficult to attribute to *H. italicum* alone, as it was usually not the only plant component tested. From the only one monotherapy trial performed by the company Aboca S.p.A., the findings were unfortunately not available. On the other hand, there are numerous studies and trials conducted with individual compounds present in *H. italicum*, which can contribute to identification of bioactive substances and elucidation of their mechanism of action. However, the findings can be misleading, as the effect of single isolated compound can be more potent due to higher concentrations achieved than in a plant, or even less manifested due to cumulative effects that occur in a compound mixture. For that reason, such findings cannot be directly extrapolated to whole plant extracts.

## 8. Conclusions

With this review, *H. italicum* has been evaluated in terms of efficacy and safety for internal use. The clinical trials provide rather more insight into the safety profile than into the efficacy, due to lack of trials performed with *H. italicum* alone. The efficacy of *H. italicum*, however, is evident from reports on traditional use, human observational studies, or in vitro research. From the data gathered, particularly the trials in young children, we conclude that the ingestion of *H. italicum* does not pose a risk to human health. Although *H. italicum* is a plant with documented immense potential in several aspects of health, it still lacks regulatory recognition. *H. italicum* could be considered for evaluation by regulatory bodies such as the Committee on Herbal Medicinal Products under the EMA based solely on its traditional use. Although ethnopharmacological reports are available, for that purpose, traditional use of *H. italicum* in European territory would have to be evaluated more thoroughly. On the other hand, to meet the well-established medicinal use criteria, novel clinical studies with *H. italicum* as a single component are needed. Based on the recent trend in *H. italicum* research, it is evident that the current interest in *H. italicum*, especially at Balkan Peninsula, is considerable and expands far beyond cosmetic applications. As various publications continue to emerge, further clinical trials can also be expected in the future.

## Figures and Tables

**Table 1:** The list of the most characteristic bioactive compounds found and investigated in *H. italicum*.

Compound Name	Chemical Structure ^1^	Extraction Solvent ^2^	Extraction Yield (from Starting Plant Material) ^3^	Main Bioactivity ^4^
Caffeic acid	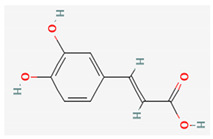	Methanol	0.0067% (NS) [38] 0.00042% (flowers) [41] 0.0057–0.015% (aerial parts or flowers) [42]	Antioxidant, anti-inflammatory and anticancer activity [44]
Chlorogenic acid	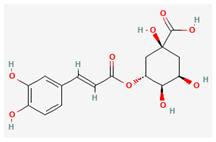	Methanol	0.104% (NS) [38] 0.045% (flowers) [41] 0.52–0.77% (aerial parts or flowers) [42]	Antibacterial [36], antiviral, antioxidant, anti-inflammatory, anticardiovascular, hypoglycemic, and anticancer activity [45]
Pinocembrin	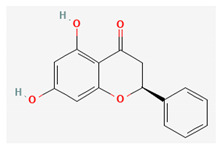	Acetone [32] Methanol [27]	NA (NS) [32] NA (aerial parts) [27]	Anti-inflammatory [29]
Quercetin	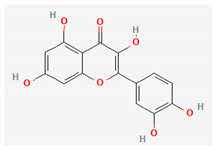	Methanol	0.015% (flowers) [41] 0.001–0.0015% (aerial parts or flowers) [42]	Antioxidant, anti-inflammatory, antimicrobial, cardioprotective, gastroprotective and anticancer activity [46]
Naringenin	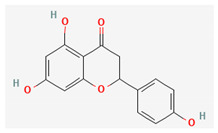	Methanol	0.023% (NS) [38]	Antioxidant, antitumor, antiviral, antibacterial, anti-inflammatory, and cardioprotective activity [47]
Gnaphaliin	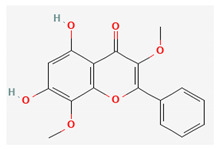	Methanol	0.03% (aerial parts) [31]	Anti-inflammatory [29]
Luteolin	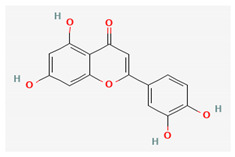	Ethanol	NA (flowers) [16]	Antiviral [24]
Tiliroside	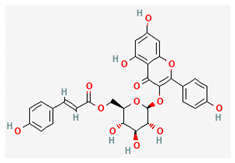	Methanol	0.0063% (aerial parts) [31] 0.0015% (flowers) [41]	Anti-inflammatory and antioxidant [29]
Arzanol	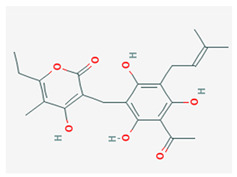	Acetone	0.078% (aerial parts) [14] 0.064% (aerial parts and flowers) [33] 0.32% (aerial parts) [37]	Anti-inflammatory [14,35], antiviral [14], antioxidant [33] and antibacterial [37]
Ursolic acid	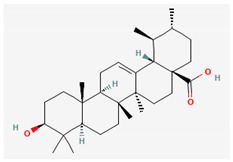	Acetone	0.40% (aerial parts) [37]	Anti-inflammatory, anticancer, antidiabetic, antioxidant and antibacterial effects [48]

^1^ Images of 2D structures of compounds were obtained from PubChem database. ^2^ Only the primary solvent used for extraction is mentioned. Solvents used in subsequent fractionation process are not listed but can be found in the referenced articles. ^3^ Extraction yields were calculated from the masses obtained after isolation procedures in response to the amount of the starting plant material. In addition, information on the plant part used in the extraction is included, if specified. ^4^ Preferentially only activity related to *H. italicum* investigation is reported, when available. NA—not available, NS—not specified.

**Table 2:** Clinical observations in humans, listed in chronological order.

Author and Date [Reference]	Observation
Santini, 1930s [49]	*H. italicum* decoction administered to patients suffering from bronchitis and asthmatic cough led to improvement of their respiratory condition and unrelated conditions such as psoriasis and arthritis.
Santini, 1930–1950 [49]	Two decades of clinical observations led to the conclusion that clinical activity of a decoction and syrup from *H. italicum* is similar to that of cortisone. Aerosolized decoction of *H. italicum* showed positive results in the use for allergic rhinitis.
Santini, 1950s [49]	Two independent clinical studies on patient with psoriasis confirmed beneficial effects of *H. italicum* treatment.
Benigni, 1950s [50]	A series of clinical studies in various Italian centers substantially confirmed the findings of Santini, showing that “Fraction H” produced using an organic solvent, could, to varying degrees, replace corticosteroids in many of their uses and their adverse side effects were thus avoided.
Vannini, 1981 [51]	*H. italicum* decoction was found to be highly efficacious in treatment of tracheo-bronchitis in a small clinical study in children.
Facino, 1988 [16]	Flavonoid fraction was applied to humans 10 min before or after exposure to UVB radiation to evaluate their photoprotective and anti-erythematous activities, respectively. The onset of the erythematous response was completely prevented and a sun protection factor of approximately 5 was provided.
Campanini, 1995 [52]	Three weeks of treatment with 5% *H. italicum* decoction led to improvement of psoriasis in all participants, with relapses observed within two months post-treatment.
Voinchet and Giraud-Robert, 2007 [54]	Two drops of *H. italicum* essential oil, orally twice a day for 10 d, followed by its topical application for 2–3 months in post-operative scars of patients submitted to a plastic surgery of the thorax led to a reduction of local inflammation, edema, bruises, and hematomas.
Granger, 2020 [55]	Night cream containing melatonin, carnosine, and *H. italicum* extract reduced skin damage caused by environmental factors and its nightly use could improve clinical signs of aging with additional skin calming benefits. Hydration and trans-epidermal water loss values were improved within 1 h of use. Wrinkle counts were reduced by up to 18.9%, and brown and UV spot numbers by 5.5% and 13.2%, respectively. Lactic acid-induced stinging was significantly reduced within 7 d of use, with 86.7% of subjects reporting that their skin felt calmer.

## Data Availability

All data is presented within the article.

## Appendix A

**Table A1:** Characteristics of included clinical trials, listed in chronological order.

Author, Year, Country	Study Type	Condition	Spec. “Italicum” Specie, Plant Parts, Compos.	Formulation, Dosage	No. of Participants, Sex	Age (y)	Duration	Main Outcome/ Results	Safety, Adverse Events
NA (Aboca S.p.A.), 2005, Italy [56]	Randomized, controlled, parallel	Post-surgery pain	Yes, flowering tops, single component	Granules for oral suspension, 2.5 g	45, m/f	18–65, >65	NA	NA	NA
Galeone, 2012, Italy [57]	Randomized, controlled	Bacterial and non-bacterial prostatitis	No, NA, comb. with other herbs	Suppository (2 g)	60, m	20–50	28 d, follow-up after 60 and 120 d	Improvement of urinary symptoms, no difference in microbiological results	Minor side effects
Varney, 2013, USA [59]	Pilot, randomized, controlled, double-blind	Mental exhaustion, moderate burnout	Yes, essential oil, mixture with two other EOs	Plastic inhaler, 2 drops of *H. italicum* EO per inhaler	14, m/f	25–45, 45–65, >65	3 weeks	Reduction in perceived level of mental fatigue/burnout	Not reported
Cohen, 2013, Israel [60]	Randomized, controlled, single-blind, parallel, multi-center	Cough associated with upper respiratory tract infections	Yes, NA, comb. with other herbs and honey	Syrup, 20 mL in three doses per day	150, m/f	2–5	4 d	Reduced cough frequency, severity, better sleep quality	Safe, well tolerated
Canciani, 2014, Italy [61]	Randomized, double-blind, multicenter, placebo-controlled	Cough associated with upper respiratory tract infections, persisting > 7 d	Yes, NA, comb. with other herbs and honey	Syrup, 4 doses per day (5 mL each)	102, m/f	3–6	8 d	The significant decrease of cough, especially evident at night	Adverse events unrelated to the treatment
Di Vico, 2019, Italy [58]	Pilot, randomized, controlled	Chronic prostatitis, chronic pelvic pain syndrome	No, NA, comb. with other herbs	Suppository (2 g), 1 supp./day	30, m	23–49	1 month	Subjective pain relief, decrease in urinary symptoms	None reported

NA—information not available, EO—essential oil, m—male, f—female, no.—number, comb.—combination, supp.—suppository, spec.—specified, compos.—composition of a product when *H. italicum* is not the single component, y—years.
